# Refractive errors among medical students in Jordan: prevalence, types and possible risk factors

**DOI:** 10.2144/fsoa-2022-0075

**Published:** 2023-03-13

**Authors:** Asem A Alqudah, Alex J Bauer, Abdelwahab Aleshawi

**Affiliations:** 1 Jordan University of Science & Technology (JUST), Irbid, 22110, Jordan; 2 Lions VisionGift, Portland, OR 97214, USA

**Keywords:** Jordan, laser refractive surgery, medical students, prevalence, refractive errors

## Abstract

**Aim:**

This study aims to determine the prevalence of refractive errors among medical students in Jordan.

**Materials & methods:**

Cross-sectional model through an online questionnaire was conducted. The questionnaire was distributed randomly to 700 medical students.

**Results:**

Females participated more than males. It was revealed that 525 (75%) of the total students were found to have a refractive error. Myopia was the most common type. About 79.0% of students have a positive family history of refractive errors which was more significant in students with refractive errors. Spectacles were the most common used method of treatment.

**Conclusion:**

The prevalence of refractive errors was high among medical students in Jordan. A positive family history was associated with students having refractive errors.

## Plain language summary

Many reports have studied refractive errors and their risk factors in different groups and ethnicities. Also, types of refractive errors were found to have a different prevalence in different age groups. Whilst refractive errors in medical students have been studied in many countries, the prevalence of refractive errors has never been studied in Jordanian medical students before. In this study, we found that myopia (with or without astigmatism) was the most common type of refractive error in medical students.

Refractive errors are a major public health problem worldwide and the most common cause of visual impairment and the second leading cause of blindness [1,2]. Previous reports have investigated the prevalence of refractive errors in different countries. A wide range of prevalence (20–80%) was found in these reports [3–6].

Different factors were found to be associated with an increased chance or risk for diagnosis of refractive errors, which include increasing educational levels, higher individual income, professional or office-related occupations, better housing, reduced sunlight exposures, prolonged near work, positive family history and greater severity of nuclear opacity [7–12].

Many previous reports also studied refractive errors and their risk factors in different groups and ethnicities. Types of refractive errors were found to have different prevalence in different age groups, with myopia being relatively common in age group 10–29 years, hyperopia has an increasing prevalence after 40 years and astigmatism has a decreasing trend with age 6–90 years [13,14]. Chen-wei pan *et al.* studied refractive errors in different racial groups in the USA and found that myopia and astigmatism were most prevalent in the Chinese population, followed by Whites, then Blacks, and least in Hispanics. Chinese subjects were found to have three-times the prevalence of myopia compared with Hispanic subjects. Hyperopia on the other hand was most common in Hispanics [15,16].

Refractive errors in medical students were also studied in many countries. In Singapore, myopia prevalence among medical students was found to be 89.8% [17]. Turkish medical students have much lower prevalence of myopia of about 32.9% [18]. Brazilian medical students have a prevalence of 70.8% [19], and Nigerian medical students had a prevalence of 79.5% [20]. In Europe, the European Eye Epidemiology (E3) Consortium concluded that the prevalence of refractive errors in Europe is distributed as follow: 30.6% for myopia, 25.2 % for hyperopia and 23.9 % for astigmatism [21].

In Jordanian medical students, the prevalence of refractive errors has never been studied before. In this report, we studied the prevalence of different refractive errors in medical students in Jordan. Moreover, possible risk factors in this group were investigated.

## Materials & methods

After obtaining the institutional review board approval, a cross-sectional design was conducted by an electronic questionnaire between March 2017 and April 2017. The included group comprised all medical students in all Jordanian medical schools. The demographics and the prevalence of different forms of refractive errors (myopia, hyperopia and astigmatism) along with their dioptric degree were assessed. In addition, questions about family history of refractive errors and the number of hours/day of electronic device use were investigated. Moreover, the corrective treatment methods used, and the reason for not performing a laser refractive surgery were collected. A form of the survey is included with this manuscript. This group of participants has similar nature of activities, duties and hours outside doors. The study was conducted in accordance with the Declaration of Helsinki and an electronic consent was obtained from all participants.

The questionnaire was constructed and conducted using a free premium account on Google Form.com. It is composed of ten systematic accurate questions. Two questions about the demographics, gender and age (the age was categorized into ‘less than 18 years’, ‘18–20 years’, ‘21–23 years’, ‘24–25 years’ and ‘more than 25 years’). The next question was the presence of refractive errors (yes or no) and type of refractive errors (myopia alone, hyperopia alone, astigmatism alone, myopia and astigmatism and hyperopia and astigmatism). Furthermore, a question about the degree of refractive errors was conducted for myopia and hyperopia (less than 1 diopter, ‘1–3 diopter’, ‘3–6 diopter’ and ‘more than 6 diopter’) and for astigmatism (less than 1 diopter, ‘1–2 diopter’, ‘2–3 diopter’ and more than 3 diopter). The age of diagnosis was also determined (‘0–5 years’, ‘5–10 years’, ‘10–15 years’, ‘15–20 years’ and ‘more than 20 years’). Moreover, family history of refractive errors in first-degree relatives was asked for all participants whether they have refractive errors or not. In addition, a question related to hours/day spent on electronic device use was determined (‘up to 2 h’, ‘2–5 h’, ‘5–8’ and ‘more than 8 h’). The treatment of refractive errors also was conducted and categorized into spectacles, contact lenses, laser refractive surgery and no correction. For those who did not perform the refractive laser surgery, the reason for that was obtained (cost, not a candidate, prefers other methods and thinking of do it later). A ‘simple random sampling method’ was used. The sampled population was distributed for five different medical schools in Jordan for all six academic levels. After construction of the survey, it was shared online on Facebook.com on official pages of medical batches from different levels and universities. These pages are constricted for the medical students. These were closed group pages in which only medical students in Jordanian universities can join.

Extracted data were entered into a spreadsheet. Statistical analysis was performed using the IBM SPSS statistical package for Windows v.22 (NY, USA). Data were expressed as frequency (percentage) for nominal data, mean ± standard deviation of the mean (SD) for normally distributed continuous variables. Statistical significance between was determined using Chi-square test for categorical variables. The sample size was confirmed using power of analysis equation at alpha level of 0.05 and power of analysis at 90%, similar anticipated incidence for both the population and the study group and found to be at least 239 participants.

## Results

A total of 700 medical students participated in the questionnaire. Females participated more than males (441 [63%] were females). The discrepancy between the proportion of males and females reflects the higher number of females accepted in the medical schools. Also, 676 (96.6%) participants were aged between both groups (18–20) and (21–23 years) which is the standard age group of medical students in Jordan. A total of 525 (75%) participants had refractive errors. Out of the 525 participants with refractive errors, 434 (82.6%) have myopia or myopia with astigmatism, 8.4% were having astigmatism only and 9% were having hyperopia or hyperopia with astigmatism. Table 1 summarizes the characteristics of the study population.

**Table 1.** General demographical characteristics of the study population.

Variables	n	Percentage (%)
Sex		
Male	259	37.0
Female	441	63.0
Age (years)		
Less than 18	7	0.1
18–20	464	66.3
21–23	212	30.3
24–25	15	2.1
More than 25	2	0.2
Presence of refractive errors		
Yes	525	75.0
No	175	25.0
Type of refractive errors		
Myopia alone	292	41.7
Myopia and astigmatism	286	40.9
Astigmatism alone	59	8.4
Hyperopia alone	34	4.9
Hyperopia and astigmatism	29	4.1
Time per day of electronic device use, n (h)		
0–2	4	0.6
2–5	169	24.1
5–8	331	47.3
More than 8	196	28.0
Method of correction (out of 525)		
Spectacles	387	73.7
Contact lens	33	6.3
Laser refractive surgery	12	2.3
No correction	93	17.7

**Figure 1.**
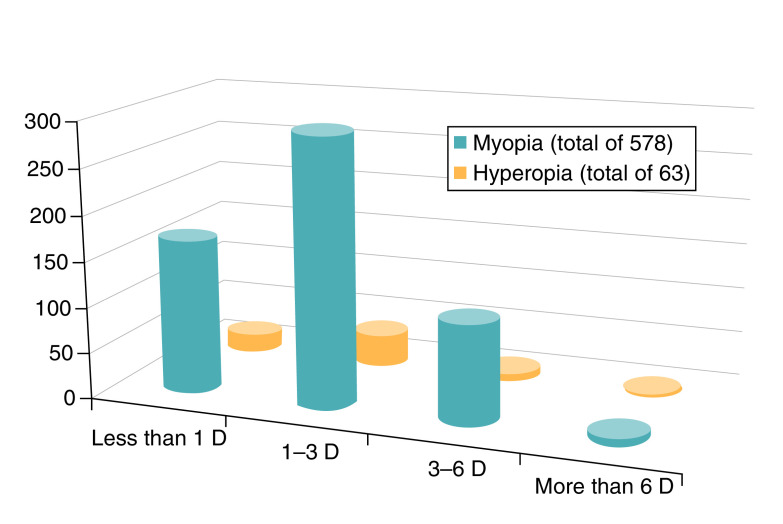
The distribution of dioptric degree of myopia and hyperopia. D: Diopter.

Most of the participant (61.4%) with refractive errors stated their refractive errors were first diagnosed between the age of 15 and 20 years, 20.8% were between 10 and 15 years and about 10% between 5 and 10 years. About half of myopic students (50.7%) have myopia within the range of (1–3) diopter, 29% have (0–1) diopter, 18.9% have (3–6) diopter and only 1.4% have a myopia of >6 diopter. Regarding hyperopia, 30% were in the range of 0–1 diopter, 55% in the range 1–3 diopter and 15% in the range 3–6 diopter Figure 1. Regarding the range of astigmatism, most participants have less than 1 D of astigmatism.

Regarding the family history, 553 participants (79.0%) stated they have at least one first-degree family member who has a refractive error. This positive family history was more clinically significant (p = 0.0008) in students with refractive errors (82.1%) compared with those without refractive errors (69.7%) Table 2. Regarding the number of hours/day spent on electronic devices, 47.2% stated they spend between 5 and 8 h/day, 28% spend >8 h/day, 24.2% 2–5 h and only 0.6% stated they spend less than 2 h/day on these devices.

**Table 2.** Relation between the presence of the refractive errors and the family history of refractive errors.

	Positive family history of RE	Negative family history of RE	p-value
Students with refractive errors	431 (82.1%)	94 (17.9%)	0.008
Students without refractive errors	122 (69.7%)	53 (30.3%)	

RE: Refractive errors.

Most of the students with refractive errors (73.8%) use glasses for correcting their refractive errors. Out of the 525 medical students who have refractive errors, only 12 (2.2%) underwent laser refractive surgery. For those students with refractive errors who did not have the laser refractive surgery (513 out of 525), a question was asked why they choose not to do so and 60% of them stated that they are fine with their current correction method and do not prefer the surgery, 27% said they are thinking of doing the laser refractive surgery later on, 7% said they were told they are not good candidates for laser refractive surgery and only 6% stated it has to do with the cost or access to the service.

## Discussion

The prevalence of refractive errors in medical students in Jordan was significantly higher than the prevalence found in general Jordanian adult population [22]. Myopia (with or without astigmatism) was the most common type of refractive error in medical students, which is supported by findings in other studies [17–20]. The age at which the refractive error was first diagnosed also supports findings in other studies [13,14]. Most of medical students (61.4%) have their refractive errors first diagnosed between the age of 15 and 20 years, this is mostly due to the natural history of myopia, and possibly due to the fact that this age range represents the period of high school education and the first 2 years of medical school education. For Jordanian medical students, these 5 years may represent the maximum hours/day of studying which may reflect prolonged near-work tasks.

The number of hours/day of electronic device use by medical students was impressive. More than 75% of students use these devices more than 5 h/day and more than a quarter use the electronic devices 8 h/day or more. This may indicate that new technology may play a role in increasing the risk for refractive errors in medical students and in the general population or increase the chance for discovering these refractive errors.

There is strong evidence that genetic factors play a part in the etiology of refractive errors [23]. In our report, family history of refractive errors was present in 553 students, representing 79.0% of the total group. There was a statistically significant difference (p = 0.0008) between family history in students with refractive errors (82.1% have positive family history) and family history in those without refractive errors (69.7% have positive family history).

This study gives supportive evidence that refractive errors have multifactorial genetic and environmental risk factors. It also supports the notion that people who are academically focused usually partake in prolonged near work; and therefore, may have a higher risk of developing refractive errors or increasing the chance of discovering such errors. Medical students in general are good representative group of people with relatively high intelligence and prolonged near work activities.

Our study also proves that despite the improvement and the widespread use of refractive surgery in the correction of refractive errors; glasses are still the most preferred modality of correction, and most medical students with refractive errors would prefer to avoid laser refractive surgery. It would be interesting to undergo a secondary survey asking medical students about their attitudes surrounding refractive surgery. It would also be interesting to see whether medical students and medical health providers in general are more or less likely to receive laser refractive surgeries than the general population.

This study is not without limitations. The nature of survey-based studies is a main limitation point in that the errors may arise from the possibility of not understanding the disease itself. It entirely depends on self-reporting from the participants rather objective clinical diagnosis. However, having a large sample of only medical students who have the basic knowledge of refractive error concepts can minimize the effect of this information bias, and we believe this large sample of medical students in a small country like Jordan is likely to be representative to the whole group.

## Conclusion

In conclusion, this is the first study to investigate the refractive errors among medical students in Jordan. It reveals that this group has high prevalence of refractive errors, with myopia being the most prevalent one. It also supports the evidence that refractive errors have multifactorial risks, both genetic and environmental.

**Summary points**

- Studying the refractive errors in medical students in certain ethnic groups is an important research area.
- It was revealed that 525 (75%) of the total students were found to have a refractive error.
- Myopia was the most common type. About 79.0% of students have a positive family history of refractive errors.

## References

[1] Naidoo KS, Leasher J, Bourne RR Global vision impairment and blindness due to uncorrected refractive error, 1990–2010. Optom. Vis. Sci. 93(3), 227–234 (2016). 26905537 10.1097/OPX.0000000000000796

[2] Bourne RR, Stevens GA, White RA Causes of vision loss worldwide, 1990–2010: a systematic analysis. Lancet Glob. Health 1(6), e339–349 (2013). 25104599 10.1016/S2214-109X(13)70113-X

[3] Pascolini D, Mariotti SP. Global estimates of visual impairment: 2010. Br. J. Ophthalmol. 96(5), 614–618 (2012). 22133988 10.1136/bjophthalmol-2011-300539

[4] Mashige KP, Jaggernath J, Ramson P Prevalence of refractive errors in the INK Area, Durban, South Africa. Optom. Vis. Sci. 93(3), 243–250 (2016). 26760577 10.1097/OPX.0000000000000771

[5] Chin MP, Siong KH, Chan KH Prevalence of visual impairment and refractive errors among different ethnic groups in schoolchildren in Turpan, China. Ophthalmic Physiol. Opt. 35(3), 263–270 (2015). 25783952 10.1111/opo.12193

[6] Soler M, Anera RG, Castro JJ Prevalence of refractive errors in children in Equatorial Guinea. Optom. Vis. Sci. 92(1), 53–58 (2015). 25360700 10.1097/OPX.0000000000000448

[7] Katz J, Tielsch JM, Sommer A. Prevalence and risk factors for refractive errors in an adult inner city population. Invest. Ophthalmol. Vis. Sci. 38(2), 334–340 (1997). 9040465

[8] Pan CW, Wong TY, Lavanya R Prevalence and risk factors for refractive errors in Indians: The Singapore Indian Eye Study (SINDI). Invest. Ophthalmol. Vis. Sci. 52(6), 3166–3173 (2011). 21296814 10.1167/iovs.10-6210

[9] Krishnaiah S, Srinivas M, Khanna RC Prevalence and risk factors for refractive errors in the South Indian adult population: The Andhra Pradesh Eye disease study. Clin. Ophthalmol. 3, 17–27 (2009). 19668540 PMC2708998

[10] Wong TY, Foster PJ, Hee J Prevalence and risk factors for refractive errors in adult Chinese in Singapore. Invest. Ophthalmol. Vis. Sci. 41(9), 2486–2494 (2000). 10937558

[11] Kim EC, Morgan IG, Kakizaki H Prevalence and risk factors for refractive errors: Korean National Health and Nutrition Examination Survey 2008–2011. PLoS ONE 8(11), e80361 (2013). 24224049 10.1371/journal.pone.0080361 PMC3818255

[12] Saad A, El Bayoumy BM. Environmental risk factors for refractive error among Egyptian schoolchildren. East. Mediterr. Health J. 13(4), 819–828 (2007). 17955764

[13] Gomez-Salazar F1, Campos-Romero A1, Gomez-Campaña H Refractive errors among children, adolescents and adults attending eye clinics in Mexico. Int. J. Ophthalmol. 10(5), 796–802 (2017). 28546940 10.18240/ijo.2017.05.23 PMC5437471

[14] Midelfart A, Kinge B, Midelfart S Prevalence of refractive errors in young and middle-aged adults in Norway. Acta Ophthalmol. Scand. 80(5), 501–505 (2002). 12390161 10.1034/j.1600-0420.2002.800508.x

[15] Pan CW, Klein BE, Cotch MF Racial variations in the prevalence of refractive errors in the United States: the multi-ethnic study of atherosclerosis. Am. J. Ophthalmol. 155(6), 1129–1138.e1 (2013). 23453694 10.1016/j.ajo.2013.01.009 PMC3759975

[16] Ying GS, Maguire MG, Cyert LA Prevalence of vision disorders by racial and ethnic group among children participating in head start. Ophthalmology 121(3), 630–636 (2014). 24183422 10.1016/j.ophtha.2013.09.036 PMC4128179

[17] Woo WW, Lim KA, Yang H Refractive errors in medical students in Singapore. Singapore Med. J. 45(10), 470–474 (2004). 15455167

[18] Onal S, Toker E, Akingol Z Refractive errors of medical students in Turkey: one-year follow-up of refraction and biometry. Optom. Vis. Sci. 84(3), 175–180 (2007). 17435530 10.1097/OPX.0b013e3180335c52

[19] Gameiro Filho AR, Aquino NMT, Pacheco EBA Knowledge in refractive surgery among medical students State University of Londrina. Rev. Bras. Oftalmol. 72(3), 172–177 (2013).

[20] Megbelayin EO, Asana UE, Nkanga DG Refractive errors and spectacle use behavior among medical students in a Nigerian medical school. J Adv. Med. Med. Res. 4(13), 2581–2589 (2014).

[21] Williams KM, Verhoeven VJ, Cumberland P Prevalence of refractive error in Europe: the European Eye Epidemiology (E(3)) Consortium. Eur. J. Epidemiol. 30(4), 305–315 (2015). 25784363 10.1007/s10654-015-0010-0 PMC4385146

[22] Mallen EA, Gammoh Y, Al-Bdour M Refractive error and ocular biometry in Jordanian adults. Ophthalmic Physiol. Opt. 25(4), 302–309 (2005). 15953114 10.1111/j.1475-1313.2005.00306.x

[23] Zadnik K1, Satariano WA, Mutti DO The effect of parental history of myopia on children's eye size. JAMA 271(17), 1323–1327 (1994). 8158816

